# Supplementary material for: Real-World Effectiveness of Mix-and-Match Vaccine Regimens against SARS-CoV-2 Delta Variant in Thailand: A Nationwide Test-Negative Matched Case-Control Study
Source: Vaccines (Basel). 2022 Jul 5;10(7):1080. doi: 10.3390/vaccines10071080 (PMC9315782; doi:10.3390/vaccines10071080)
Supplement: Supplementary file 1 [file vaccines-10-01080-s001.zip › vaccines-1760407-supplementary.pdf]

Supplementary Material

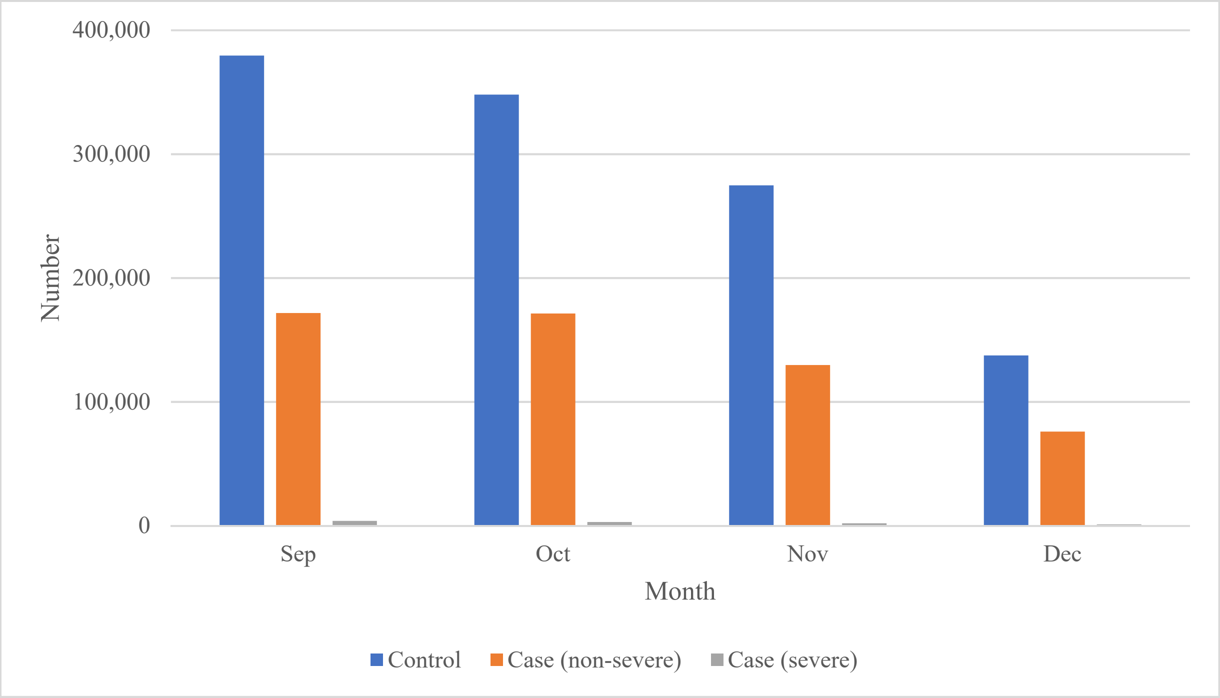

Figure S1. Number of cases and controls over months.

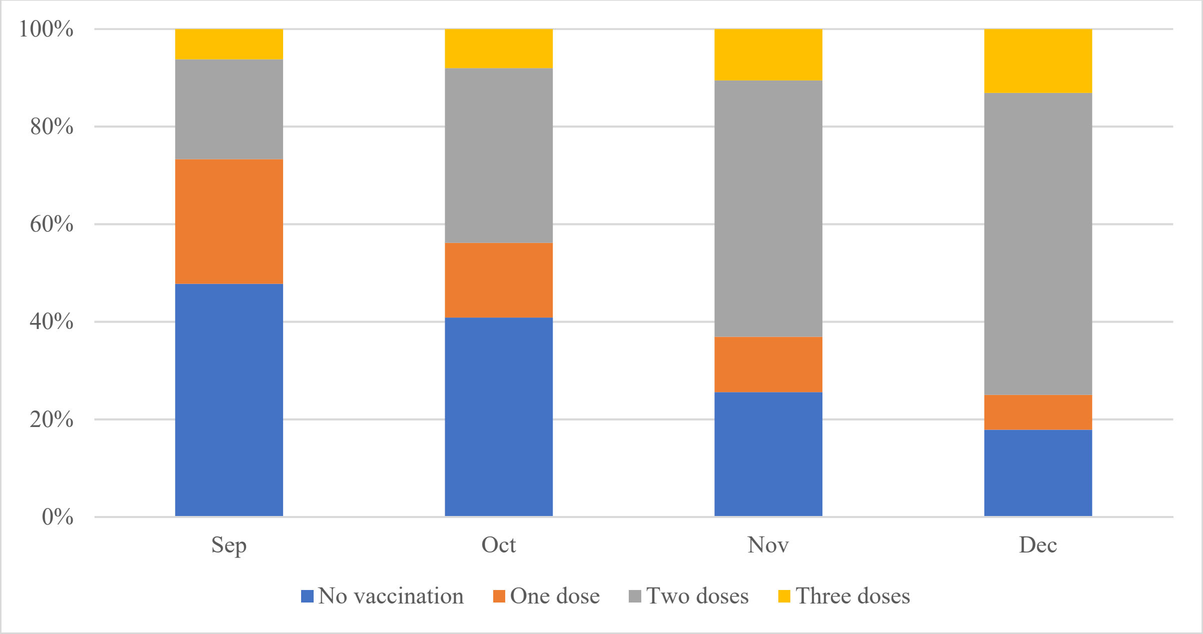

Figure S2. Proportion of each vaccination status over months.

Table S1. Descriptive statistics of the time lag between laboratory collection date and last vaccination date for the vaccinees whose laboratory collection date occurred at least 90 days after the last vaccination date.

Regimen	Mean (standard deviation)	Median (P25, P75)
Two-dose		
• BNT162b2+BNT162b2	99 (7)	98 (93, 104)
• ChAdOx1+ChAdOx1	107 (19)	101 (95, 112)
• CoronaVac+CoronaVac	126 (18)	122 (104, 142)
• ChAdOx1+BNT162b2	107 (12)	104 (96, 116)
• CoronaVac+ChAdOx1	108 (15)	105 (96, 117)
• CoronaVac+BNT162b2	108 (15)	105 (96, 118)
Three-dose		
• CoronaVac+CoronaVac+ChAdOx1	116 (101, 133)	118 (19)
• CoronaVac+CoronaVac+BNT162b2	108 (98, 122)	110 (14)
